# m6A methyltransferase METTL3-induced lncRNA SNHG17 promotes lung adenocarcinoma gefitinib resistance by epigenetically repressing LATS2 expression

**DOI:** 10.1038/s41419-022-05050-x

**Published:** 2022-07-28

**Authors:** Heng Zhang, Shao-Qiang Wang, Li Wang, Hang Lin, Jie-Bo Zhu, Ri Chen, Lin-Feng Li, Yuan-Da Cheng, Chao-Jun Duan, Chun-Fang Zhang

**Affiliations:** 1 Department of General Thoracic Surgery, Xiangya Hospital, Central South University, Changsha, 410008 Hunan Province P. R. China; 2 Xiangya Lung Cancer Center, Xiangya Hospital, Central South University, Changsha, 410008 Hunan Province P. R. China; 3 Hunan Engineering Research Center for Pulmonary Nodules Precise Diagosis&Treatment, 410008 Changsha, Hunan Province P. R. China; 4 National Clinical Research Center for Geriatric Disorders, Xiangya Hospital, Changsha, 410008 Hunan Province P. R. China; 5 Department of Thoracic Surgery, Affiliated Hospital of Jining Medical University, Jining Medical University, Jining, 272029 Shandong Province P. R. China; 6 Department of Thoracic Surgery, The Second Xiangya Hospital of Central South University, Changsha, 410011 Hunan Province P. R. China; 7 Hunan Key Laboratory of Early Diagnosis and Precise Treatment of Lung Cancer, The Second Xiangya Hospital of Central South University, Changsha, 410011 Hunan Province P. R. China

**Keywords:** Cell death, Lung cancer

## Abstract

Gefitinib has been widely applied for the treatment of lung adenocarcinoma (LUAD). However, the long-term application of gefitinib usually leads to acquired drug resistance in tumour patients, resulting in clinical treatment failure. Small nucleolar host gene 17 (SNHG17) has been shown to play a regulatory role in LUAD progression. Nevertheless, the role of SNHG17 in LUAD gefitinib resistance remains elusive. The expression pattern of SNHG17 was examined in tissues and cell lines of gefitinib-sensitive and gefitinib-resistant LUAD, respectively. Gain- and loss-of-function experiments were employed to assess the biological functions of SNHG17 in cell proliferation and apoptosis, as well as aggressive phenotypes of LUAD cells. MeRIP-qPCR and colorimetric quantificational analysis were performed to detect m6A modifications and contents. Fluorescence in situ hybridisation (FISH) and subcellular fractionation analysis were used to reveal the distribution of SNHG17. RIP and ChIP assays were performed to further validate the SNHG17/EZH2/LATS2 regulatory axis. A xenograft tumour growth assay was conducted to evaluate the role of SNHG17 in LUAD gefitinib resistance in vivo. SNHG17 was upregulated in gefitinib-resistant LUAD tissues and cell lines. Functional assays showed that SNHG17 aggravated the malignant phenotypes of gefitinib-resistant LUAD cells. In addition, METTL3-mediated N^6^-methyladenosine modification could induce the upregulation of SNHG17by stabilising its RNA transcript. Mechanistically, SNHG17 epigenetically repressed the expression of LATS2 by recruiting EZH2 to the promoter region of LATS2. The regulatory role of the SNHG17/EZH2/LATS2 axis in LUAD gefitinib resistance was further supported in vivo. Collectively, our findings suggested that SNHG17 induced by METTL3 could promote LUAD gefitinib resistance by epigenetically repressing LATS2 expression.

## Introduction

Lung cancer, is one of the leading causes of tumour-related death in the world, leading to more than one million deaths per year [1]. As the most common subtype of lung cancer, lung adenocarcinoma (LUAD) is reported to account for more than 40% of all cases [2]. Despite tremendous advances in screening and treatment strategies for LUAD over the past few decades, its mortality rate continues to rise, particularly in developing economies [3]. During previous decades, multiple mutations and rearrangements of LUAD driver genes have been identified, and the mutation in the epidermal growth factor receptor (EGFR) is the most well-characterised [4]. The application of EGFR tyrosine kinase inhibitors (EGFR-TKIs), such as gefitinib and erlotinib, has shown tremendous benefits in LUAD patients harbouring activating EGFR mutations [5]. Nevertheless, acquired resistance usually develops in approximately one-third of subjects after a median of 9–13 months of drug usage [6], resulting in therapy failure. Thus, it is essential to elucidate the mechanisms of EGFR-TKI resistance.

Long noncoding RNAs (lncRNAs) have been demonstrated to participate in numerous tumour-associated processes, including cell proliferation, invasion, autophagy and drug resistance [7]. Increasing evidence revealed the involvement of lncRNAs in EGFR-TKI resistance in lung cancer patients with EGFR mutations [8]. For example, the overexpression of LINC00665 has been reported to induce gefitinib resistance by recruiting EZH2 to activate the PI3K/AKT pathway [9]. Recently, lncRNA UCA1 has also been found to induce gefitinib resistance in NSCLC by inhibiting CDKN1A expression [10]. SNHG17 was a differentially expressed lncRNA that was first discovered as an oncogene in colorectal cancer (CRC) by Ma et al. in 2017 [11]. Afterwards, an oncogenic role of the lncRNA SNHG17 was also discovered in various human cancers, such as gastric cancer, glioma and breast cancer [12–14]. Interestingly, lncRNA SNHG17 was reported to facilitate LUAD progression by acting as a sponge for miR-485-5p [15]. However, the role of lncRNA SNHG17 in the gefitinib resistance of LUAD has not been clearly investigated.

*N*^6^-Methyladenosine (m6A) is a common type of mRNA internal modification that is involved in the regulation of gene expression by modulating RNA processing, localisation, translation and eventual decay [16, 17]. m6A modification is mainly regulated by three types of regulators, methyltransferases (METTL3, METTL14 and RBM15), demethylases (ALKBH5 and FTO), and RNA reader proteins (EIF3, YTHDC1 and HNRNPC), which are also known as “writers”, “erasers” and “readers”, respectively [18]. m6A-mediated RNA modification is co-transcriptionally installed by the methyltransferase complex, which consists of the METTL3 subunit and other accessory subunits [16]. As one of the most studied methyltransferases, METTL3 has been reported to be involved in the progression of a variety of cancers [19, 20]. In LUAD, METTL3 could facilitate tumour growth and ferroptosis by promoting the m6A modification of SLC7A11 [21]. In recent decades, increasing evidence has shown the involvement of m6A modification in the regulation of lncRNAs [22]. However, whether m6A modification is involved in the translation and stabilisation of lncRNA SNHG17 remains largely unknown.

Large tumour suppressor kinase 2 (LATS2) is a well-known tumour repressor protein found in drosophila [23]. It is the core component of the Hippo cascade that can regulate various biological events, such as cell proliferation and apoptosis [24]. For example, the restoration of LATS2 repressed the survival of retinoblastoma cells by modulating the Hippo-YAP signalling pathway [25]. However, there are few reports on the roles and mechanisms of LATS2 in LUAD. Evidences have shown that the LATS2 promoter is usually hypermethylated in a variety of human tumours, such as lung cancer (~79%) [26], breast cancer (~50%) [27], and astrocytoma (~71%) [28], implying an important role of LATS2 hypermethylation in regulating biological events in tumour cells. However, whether LATS2 has a regulatory correlation with lncRNA SNHG17 remains unknown.

Herein, we showed that SNHG17 (NR_015366.5) was highly expressed in both gefitinib-resistant tissues and cell lines, and increased SNHG17 was proven to exacerbate LUAD malignant phenotypes. In addition, METTL3 was verified to be a crucial m6A methyltransferase to maintain the stability of SNHG17. Mechanistically, SNHG17 could epigenetically suppress LATS2 by binding to EZH2, subsequently promoting LUAD progression. In summary, our findings suggested that SNHG17 activated by METTL3 could promote LUAD gefitinib resistance by epigenetically repressing LATS2 expression, which provided some promising therapeutic targets for the treatment of gefitinib-resistant LUAD patients.

## Materials and methods

### Tissue collection and cell lines

A total of 36 LUAD tumour tissues and 36 normal control lung tissues were obtained from Xiangya Hospital, Central South University. All tissue samples were collected by lung biopsy or bronchoscopic biopsy and pathologically diagnosed by pathologists. The enroled LUAD patients were only administered gefitinib for ~1 year prior to biopsy. According to the tumour size, CR and the changes in serum biomarkers (CEA, CA125, Cyfra21-1 and NSE), 13 LUAD patients were defined as the gefitinib-sensitive group and 23 LUAD patients were defined as the gefitinib-resistant group. The investigator was blinded to the group allocation during the experiment. The study was approved by the ethical review board of Xiangya Hospital, Central South University, and informed consent was obtained from all subjects.

The human LUAD cell lines PC9 and A549, as well as the human lung epithelial cell line BEAS-2B, were obtained from American Type Culture Collection (ATCC; Manassas, VA, USA). All cell lines were identified by STR profiling and without contamination with mycoplasma. The cells were cultured in RPMI 1640 medium (Invitrogen, Carlsbad, CA, USA) and exposed to 95% air and 5% CO_2_. No mycoplasma infection was found in these cell lines. Gefitinib-resistant PC9 (PC9/GR) and A549 (A549/GR) cell lines were generated by chronic exposure to gefitinib. In brief, PC9 and A549 cells were exposed to gefitinib (10 nmol/L) for 48 h. After washing, the cells were further cultured in a drug-free culture medium until the cells reached 80% confluence. Afterwards, the cells were again exposed to increasing concentrations of gefitinib, and the process was repeated. Finally, the cells that survived stably in 1 μmol/L gefitinib were collected and identified as gefitinib-resistant cells.

### Cell transfection

Short hairpin RNAs (shRNAs) specifically targeting SNHG17 (shSNGH17) and the corresponding negative controls (sh-NC) were obtained from Keygen Biotech (Jiangsu, China). The shRNAs were cloned into a lentivirus vector (pHBLV-U6-MCS-CMV-ZsGreen-PGK-Puro) to construct lv-sh-SNGH17 vectors (lv-sh-SNHG17#1 and lv-sh-SNHG17#2). The recombinant lentivirus vectors were transfected to HEK293T cells for viral plasmid replication. The viral supernatants were collected 48 h after infection. The full sequences of SNHG17 and EZH2 were inserted into the pLCDH-ciR vector (Geenseed Biotech, Guangzhou, China), and then transfected into LUAD cells to generate stably transfected cells. siRNA targeting EZH2 (siEZH2) and its negative control (siNC) were purchased from Sangon Biotech (Shanghai, China). pcDNA3.1-LATS2 and pcDNA3.1-METTL3 as well as empty pcDNA3.1 vectors were purchased from Sangon Biotech. For cell transfections, the above RNAs were transfected using Lipofectamine 3000 (Invitrogen). Post-transfection for 48 h, the transfection efficiency was measured by qRT-PCR.

### RNA extraction and quantitative reverse transcription PCR (qRT-PCR)

Total RNA from LUAD tissues and cell lines was prepared by using TRIzol Reagent (Thermo Fisher Scientific, Waltham, MA, USA). RNA was then reverse transcribed into cDNA using PrimerScript RT Reagent Kit (Takara, Dalian, China). qRT-PCR was conducted using TB Green Premix Ex Taq (Takara) on an ABI7500 Fast Real-Time PCR System (PE Applied Biosystems, Thermo Fisher Scientific). The relative expression levels of genes were measured using the 2^−ΔΔCt^ method. The sequences of the primers used in this study was listed in Table 1.

**Table 1:** The primers sequences of qRT-PCR.

Gene	Forward/Reverse	Sequence (5′ -3′)
SNHG17	Forward	AGGGGAAGCAAGGTGAAAGT
	Reverse	ATCCCAGATCACCAACTCCA
METTL3	Forward	GAGTGCATGAAAGCCAGTGA
	Reverse	CTGGAATCACCTCCGACACT
P27	Forward	TCTACTGCGTGGCTTGTCAG
	Reverse	TGTATTTGGAGGCACAGCAG
HMGA2	Forward	ACTTCAGCCCAGGGACAAC
	Reverse	TCCAGTGGCTTCTGCTTTCT
LATS2	Forward	ACAAGATGGGCTTCATCCAC
	Reverse	CTCCATGCTGTCCTGTCTGA
KLF2	Forward	GCCGTCCTTCTCCACTTTC
	Reverse	GGGTTCGGGGTAATAGAACG
TIMP2	Forward	GATGCACATCACCCTCTGTG
	Reverse	GTGCCCGTTGATGTTCTTCT
U6	Forward	CTCGCTTCGGCAGCACA
	Reverse	AACGCTTCACGAATTTGCGT
GAPDH	Forward	AGGTCGGAGTCAACGGATTT
	Reverse	TGACGGTGCCATGGAATTTG

### Cell Counting Kit-8 (CCK-8) assay of IC_50_

The treated cells were inoculated into 96-well plates (2 × 10^4^ cells/well). After discarding the culture medium, 200 µL of culture medium containing different concentrations were added to each well. CCK-8 (20 µl, Dojindo, Japan) reagent was then added to each well and incubated for 4 h. The optical density was examined by a microplate reader at 450 nm. IC_50_ was determined as the concentration of gefitinib showing 50% cell growth inhibition.

### Flow cytometry analysis

After indicated treatments, cells were harvested in the exponential growth phase and incubated with FITC-Annexin V and propidium iodide using a FITC-Annexin V apoptosis detection kit (BD Biosciences, San Diego, CA, USA). Cell apoptosis was then analysed using a flow cytometer (FACScan, BD Biosciences). A BD Cycle Test Plus DNA Reagent Kit (BD Biosciences) was adopted for cell cycle analysis following the manufacturer’s protocol.

### EdU incorporation assay

In brief, treated cells (2 × 10^4^ cells/well) were seeded into 24-well plates, and newly synthesised DNA was tested with an EdU assay kit (Ribobio, Guangzhou, China) according to the manufacturer’s instructions. In brief, the cells were cultured at 37 °C for 24 h and then incubated with an EdU reagent (50 µmol/L) for another 2 h. The cells were washed with PBS and then fixed in 4% paraformaldehyde for 0.5 h, followed by permeabilization in 0.5% Triton X-100 for 1 h. DAPI was used to stain the nuclei of the cells. After staining, the cells were observed and imaged under a confocal microscope (Olympus, Japan).

### Colony formation assay

Transfected cells were seeded into 6-well plates at a density of 1000 cells/well and maintained for 2 weeks at 37 °C. Afterwards, visible colonies were stained with 0.1% crystal violet after fixation in 4% paraformaldehyde for 1 h, and the plates were imaged under a microscope (Olympus) at 20× magnification.

### Transwell assay

Transwell chambers (8 μm, Millipore, Billerica, MA, USA) with or without Matrigel were used to evaluate the invasive and migratory abilities of treated cells. For invasion and migration analysis, treated cells (2 × 10^4^) were cultured in a serum-free culture medium and seeded into the upper chamber, and a 500 μL culture medium containing 20% FBS was placed in the lower chamber. After 48 h, cells in the upper chamber were removed, and cells in the lower chamber were stained with crystal c violet (0.1%). Then, the migrating and invading cells were observed and captured under a microscope (Olympus).

### Western blot

RIPA buffer (Beyotime, Shanghai, China) was used to isolate total proteins from treated cells and tissues. After determining the protein concentration, 50 micrograms of proteins were separated by 10% SDS–PAGE. Afterwards, the target proteins were transferred to PVDF membranes and followed by blocked in 5% low-fat milk for 2 h. Then, the membranes were incubated with the primary antibodies against E-cadherin (1:200, ab40772, Abcam, Cambridge, UK), N-cadherin (1:10000, ab76011, Abcam), Vimentin (1:2000, ab137321, Abcam), Snail (1:1000, ab216347, Abcam), LATS2 (1:2000, ab110780, Abcam), EZH2 (1:25, ab227648, Abcam) and GAPDH (1:5000, ab8245, Abcam). After washing with PBS for three times, the membranes were stained with the indicated HRP-conjugated secondary antibody for 2 h. Enhanced chemiluminescence reagent (Millipore) was employed to detect the bands, and the band intensities were analysed by ImageJ software.

### Immunofluorescence staining

Treated cells were harvested and fixed with 4% paraformaldehyde for 15 min, and further permeabilized using 0.2% Triton X-100 for 10 min. After washing with PBS three times, cells were incubated with primary antibodies against E-cadherin (1:500, ab40772, Abcam) and N-cadherin (1:200, ab18203, Abcam) in PBS overnight. After washing with PBS three times, the cells were stained with anti-mouse IgG H&L secondary antibody (1:200, ab150113, Abcam) for 2 h. The cells were imaged using a confocal microscope (Olympus).

### Methylated RIP-qPCR (MeRIP-qPCR)

PolyA+ RNAs from treated gefitinib-sensitive and gefitinib-resistant LUAD cells were isolated and subjected to m6A ribonucleoprotein immunoprecipitation. Protein G Dynabeads (Thermo Fisher Scientific) were incubated with anti-m6A (Millipore) or anti-IgG (Millipore) for 24 h. After washing with IPP buffer three times, PolyA+ RNA (100 ng), DTT (1 mM), and RNase were added to the beads, the mixture volume was brought to 500 μl with IPP buffer and the mixture was incubated for 4 h. The beads were washed again with IPP buffer five times, and then the m6A RNA was eluted by tumbling in *N*6-methyladenosine-5′ -monophosphate sodium salt (CHEM-IMPEX INT’L INC, USA). Next, the supernatant was added to TRIzol-LS, and RNA was isolated following the manufacturer’s instructions. For qRT-PCR, m6A PolyA + RNA was reverse transcribed into cDNA using the iScript cDNA synthesis kit (Bio-Rad, CA, USA). Then, cDNA samples were subjected to qRT-PCR.

### m6A RNA methylation quantification

An EpiQuik m6A RNA Methylation Quantitation Kit (Epigentek, NY, USA, colorimetric method) was adopted for the quantification of m6A RNA methylation level in PC9/GR and A549/GR cells following the manufacturer’s protocols.

### RNA stability

To measure RNA stability, treated cells were cultured in 24-well plates for 24 h and then exposed to actinomycin D (5 μg/ml) for 0, 3 and 6 h. Afterwards, the cells were harvested for extraction of total RNA by using TRIzol reagent (Thermo Fisher Scientific). Then, the remaining SNHG17 level was detected by qRT-PCR.

### Subcellular fractionation

The Nuclear and Cytoplasmic Extraction kit (Thermo Fisher Scientific) was adopted to isolate the cytoplasmic and nuclear parts of PC9/GR and A549/GR cells. Then, RNA samples were extracted and quantified by qRT-PCR analysis as described above. U6 was applied as a nuclear control, and GAPDH was applied as cytoplasmic control.

### Fluorescence in situ hybridisation (FISH)

The specific Cy3-labelled lncRNA SNHG17 probe (-ATGGACAGAGGGATGCGAGGCT-) was obtained from RiboBio. Treated PC9/GR cells were fixed with 4% paraformaldehyde and then dehydrated with a 70 to 100% ethanol series. Afterwards, the cells were incubated with the specific probe in a hybridisation buffer after air-drying. Hoechst dye was used to detect cell nuclei. The cells were imaged by a confocal microscope (Olympus).

### RNA immunoprecipitation (RIP)

In brief, treated cells were lysed with RIP lysis buffer. The cell extract was then incubated with magnetic beads conjugated with antibodies against EZH2 (1:100, ab227648, Abcam), LSD1 (1:200, ab224270, Abcam) and IgG (1:200, ab172730, Abcam) for 8 h at low temperature (4 °C). After washing with PBS, the beads were incubated with Proteinase K, followed by the detection of lncRNA SNHG17 using qRT-PCR.

### Chromatin immunoprecipitation (ChIP) assay

ChIP was performed using an EZ ChIP Chromatin Immunoprecipitation Kit (Millipore). Briefly, the cross-linked chromatin was sonicated into small fragments, and then these fragments were precipitated by anti-EZH2 (b227648, Abcam) and anti-H3K27me3 (ab6002, Abcam) from the cell lysates. Anti-IgG (ab172730, Abcam) was applied as a negative control. Finally, the enrichment of LATS2 mRNA in the precipitated complex was quantified using the qRT-PCR assay.

### Mouse xenograft model

Animal manipulations were performed in accordance with the National Institute of Health guidelines with the approval of the Ethics Committee of Xiangya Hospital, Central South University. Nude mice (male, 6–8 weeks old) supplied by Slac-Jingda Laboratory Animal (Hunan, China) were maintained in specific pathogen-free conditions. These mice were randomly divided into the following groups: sh-NC, sh-SNHG17#1, sh-NC + Gef and sh-SNHG17#1+Gef (*n* = 8 per group). All experimenters were blinded to the group allocation during the experiment. PC9/GR and A549/GR cells stably transfected with lv-sh-NC or lv-sh-SNHG17 were resuspended in PBS at a density of 2 × 10^6^ cells/mL. The indicated cell suspensions were then injected into the right flanks of the mice. Four days after inoculation, gefitinib was administered to the mice by oral gavage (25 mg/kg, 5 days per week). Tumour volumes were detected every 4 days using the calculation formula: (length × width^2^/2). After infection for 28 days, the mice were euthanized and tumour tissues were collected for tumour weight and further subsequent experiments.

### Statistical analysis

Three independent replicates were performed for all cell experiments and for each clinical sample, and eight replicates were performed for each animal-level experiment. Statistical analyses were conducted on GraphPad Prism (Version 6.0, USA). Data were presented as the mean ± standard deviation (SD). All data were accorded with the requirement of normal distribution and homogeneity of variance. Intergroup comparisons were performed with unpaired student’s *t*-test, and multiple comparisons were done by one-way ANOVA followed by Tukey’s post hoc test. *P* < 0.05 was considered statistically significant.

## Results

### LncRNA SNHG17 was elevated in gefitinib-resistant LUAD

**Fig. 1:**
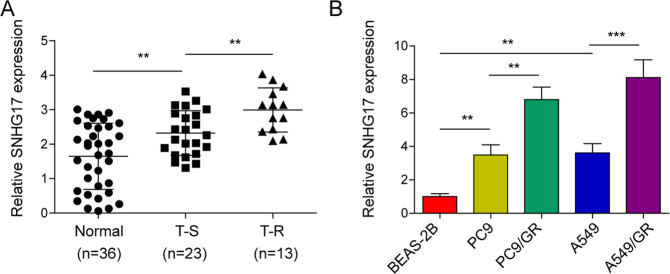
LncRNA SNHG17 was elevated in gefitinib-resistant LUAD. **A** qRT-PCR analysis of lncRNA SNHG17 in normal (*n* = 36), gefitinib-sensitive (*n* = 23), and gefitinib-resistant (*n* = 13) LUAD tissues. **B** qRT-PCR detection of lncRNA SNHG17 in LUAD cells (PC9 and A549) and gefitinib-resistant LUAD cell lines (PC9/GR, A549/GR). BEAS-2B was used as a normal control. The data were expressed as the mean ± SD of *n* = 3 experiments. **P < 0.01; ***P < 0.001.

To determine whether the lncRNA SNHG17 plays a role in gefitinib resistance in LUAD, qRT-PCR was used to detect the lncRNA SNHG17 level in normal, gefitinib-sensitive and gefitinib-resistant tissue samples. LncRNA SNHG17 was revealed to be dramatically elevated in gefitinib-sensitive LUAD tissue samples compared to normal samples (Fig. 1A). In addition, the relative level of lncRNA SNHG17 in the gefitinib-resistant group was significantly higher than that in the gefitinib-sensitive group (Fig. 1A). We also examined the expression of lncRNA SNGH17 in LUAD cells. The results indicated that the lncRNA SNHG17 level in LUAD cell lines (PC9 and A549) was dramatically upregulated compared to that in the BEAS-2B cell line (Fig. 1B). Meanwhile, we found that the expression of lncRNA SNHG17 was significantly higher in gefitinib-resistant LUAD cell lines (PC9/GR, A549/GR) than in the parent cell lines (PC9 and A549) (Fig. 1B). The increased lncRNA SNGH17 in gefitinib-resistant LUAD suggested that it may be involved in the regulation of gefitinib resistance in LUAD.

### LncRNA SNHG17 regulated proliferation, apoptosis and gefitinib resistance in LUAD cells

**Fig. 2:**
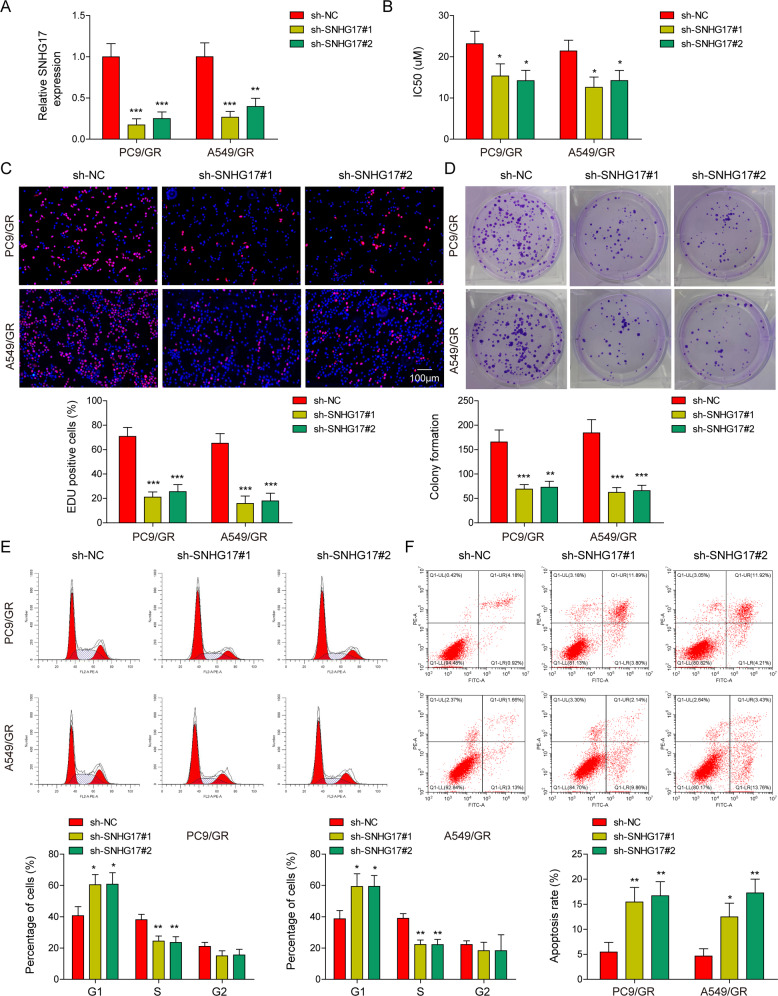
LncRNA SNHG17 knockdown repressed proliferation while promoting apoptosis in PC9/GR and A549/GR cells. **A** Two shRNAs (sh-SNHG17#1 and sh-SNHG17#2) were generated to silence lncRNA SNHG17 expression in PC9/GR and A549/GR cells. **B** IC_50_ value of gefitinib in PC9/GR and A549/GR cells after 48 h of transfection with sh-NC, sh-SNHG17#1 and sh-SNHG17#2. **C** EdU staining and **D** colony formation assays were applied to estimate the impacts of sh-NC, sh-SNHG17#1 and sh-SNHG17#2 on proliferation in PC9/GR and A549/GR cells. Flow cytometry analysis of the cell cycle (**E**) and **F** apoptosis was performed after 24 h of transfection with sh-NC, sh-SNHG17#1 and sh-SNHG17#2 in PC9/GR and A549/GR cells. Data were representative images or were expressed as the mean ± SD of *n* = 3 experiments. *P < 0.05, **P < 0.01, ***P < 0.001.

Subsequently, the role of SNHG17 in LUAD gefitinib resistance was studied with functional loss- and gain- experiments. Two specific shRNAs targeting lncRNA SNHG17 (lv-sh-SNHG17#1 and lv-sh-SNHG17#2) were transfected into PC9/GR and A549/GR cells, and SNHG17-overexpression vectors were transfected into PC9 and A549 cells. After transfection, the qRT-PCR results indicated a remarkable downregulation of SNHG17 in PC9/GR and A549/GR cells and an obvious increase in SNHG17 in PC9 and A549 cells (Fig. 2A and Fig. S1A). The CCK-8 assay indicated that SNHG17 knockdown decreased the IC_50_ value of PC9/GR and A549/GR cells, while SNHG17 overexpression enhanced the IC_50_ value of gefitinib in PC9 and A549 cells (Fig. 2B and Fig. S1B). In addition, EdU staining and colony formation assays showed that SNHG17 knockdown dramatically repressed the cell proliferation capacity in PC9/GR and A549/GR cells, and SNHG17 upregulation greatly increased cell viability and proliferation in PC9 and A549 cells (Fig. 2C, D and Fig. S1C, D). The effects of lncRNA SNGH17 on cell cycle and apoptosis were also evaluated using flow cytometry analysis. The results indicated that lncRNA SNHG17 knockdown significantly increased the cell number in G0/G1 phases while decreasing the cell number in the S phase in PC9/GR and A549/GR cells, and lncRNA SNHG17 upregulation exhibited the opposite effects in PC9 and A549 cells (Fig. 2E and Fig. S1E). Moreover, lncRNA SNHG17 silencing greatly enhanced the apoptosis rate in PC9/GR and A549/GR cells, and lncRNA SNHG17 overexpression further reduced the cell apoptosis rate in PC9 and A549 cells (Fig. 2F and Fig. S1F). These findings indicated that the lncRNA SNHG17 plays an important role in regulating proliferation, apoptosis and gefitinib resistance of LUAD cells.

### LncRNA SNHG17 regulated the migration, invasion and EMT of LUAD cells

**Fig. 3:**
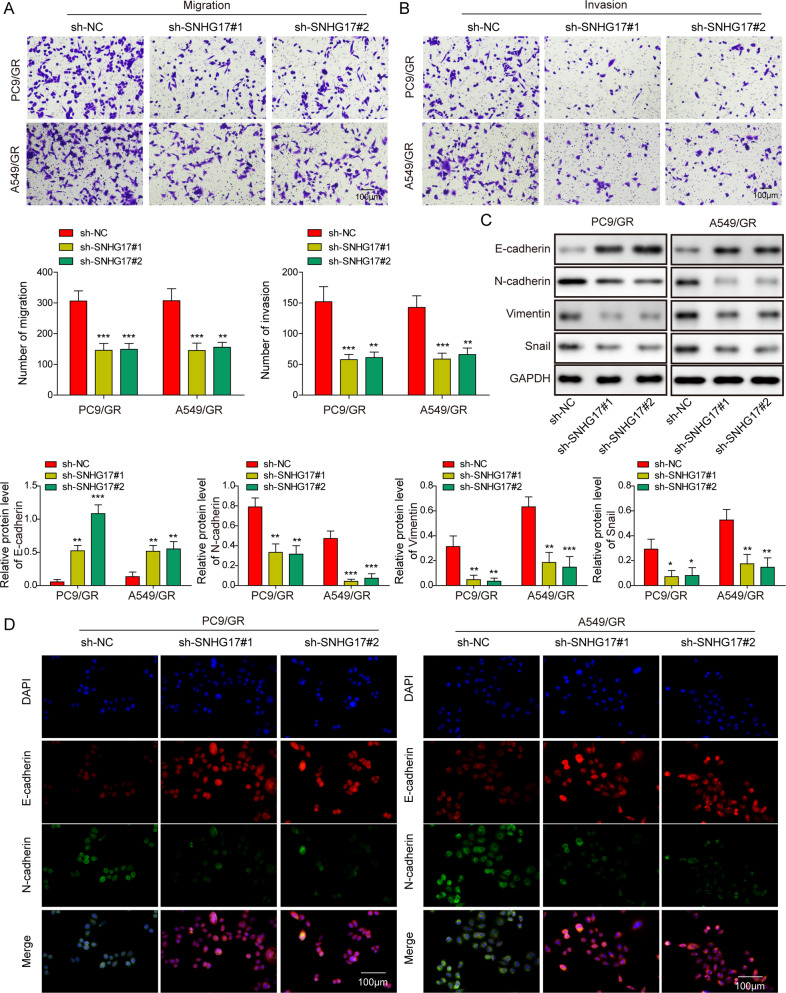
LncRNA SNHG17 knockdown repressed migration, invasion and EMT in PC9/GR and A549/GR cells. **A**, **B** After transfection for 24 h, PC9/GR and A549/GR cells were subjected to migration and invasion analysis using a transwell assay. **C** Protein expression of E-cadherin, N-cadherin, Vimentin and Snail in PC9/GR and A549/GR cells were tested via western blot. **D** Immunofluorescence analysis of E-cadherin and N-cadherin in treated PC9/GR and A549/GR cells. Data were representative images or were expressed as the mean ± SD of *n* = 3 experiments. *P < 0.05, **P < 0.01, ***P < 0.001.

Next, the impacts of lncRNA SNHG17 on the migration, invasion and EMT of LUAD/GR and LUAD cells were estimated. In transwell assay, we observed significant repression of migration and invasion in PC9/GR and A549/GR cells transfected with sh-SNHG17#1 and sh-SNHG17#2 compared to the sh-NC groups; in contrast, lncRNA SNHG17 overexpression greatly enhanced cell migration and invasion in PC9 and A549 cells (Fig. 3A, B and Fig. S2A,B). Additionally, western blot and immunofluorescence analysis showed that lncRNA SNHG17 knockdown dramatically upregulated the expression of E-cadherin and decreased the expression of N-cadherin, Vimentin and Snail in PC9/GR and A549/GR cells, while lncRNA SNHG17 overexpression had the opposite effect in PC9 and A549 cells (Fig. 3C, D and Fig. S2C, D**)**. Overall, these findings illustrated that lncRNA SNHG17 could contribute to the migration, invasion and EMT of LUAD and LUAD/GR cells.

### METTL3 increased the abundance of m6A and enhanced the stability of lncRNA SNHG17

**Fig. 4:**
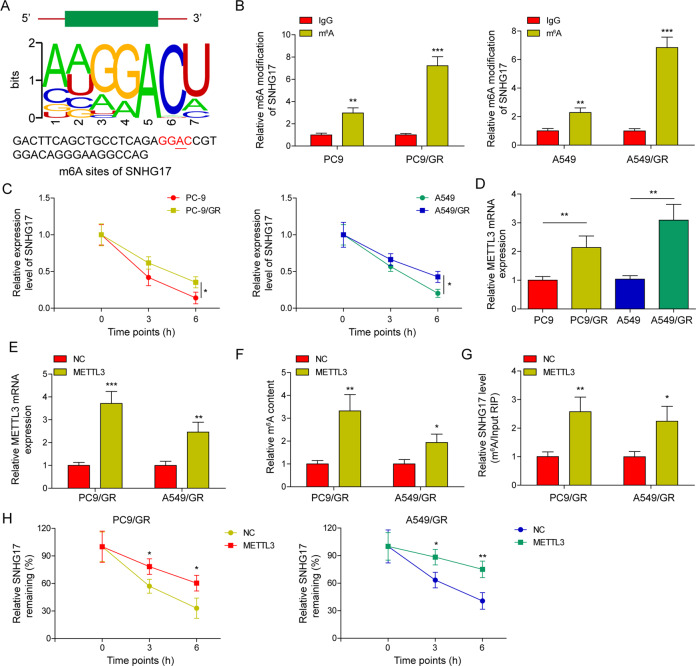
METTL3 increased the abundance of m6A and enhanced the stability of lncRNA SNHG17. **A** m6AVar predicted potential m6A modification on the lncRNA SNHG17 sequence. **B** MeRIP-qPCR was performed to detect m6A expression in PC9 and A549 cells as well as in the corresponding gefitinib-resistant cells. **C** PC9, A549, PC9/GR and A549/GR cells were treated with actinomycin D for 0, 3 and 6 h, followed by the detection of lncRNA SNHG17 expression using qRT-PCR. **D** METTL3 expression in PC9, A549, PC9/GR and A549/GR cells. **E** qRT-PCR analysis of METTL3 mRNA level in PC9/GR and A549/GR cell lines after transfection with METTL3. **F** The m6A modification of PC9/GR and A549/GR cells transfected with METTL3 was examined by colorimetric quantificational analysis. **G** MeRIP-qPCR was performed to detect the m6A modification of lncRNA SNHG17 in METTL3-overexpressing PC9/GR and A549/GR cells. **H** RNA stability examination was performed to assess the influences of METTL3 overexpression on the stability of lncRNA SNHG17 in PC9/GR and A549/GR cells. Data were expressed as the mean ± SD of *n* = 3 experiments. *P < 0.05, **P < 0.01, ***P < 0.001.

We observed a potential m^6^A modification on the lncRNA SNHG17 sequence (Fig. 4A) using an online tool (m6AVar, http://m6Avar.renlab.org/). The results from MeRIP-qPCR indicated that the m6A modification of SNHG17 in PC9/GR and A549/GR cells was significantly increased compared with that in PC9 and A549 cells (Fig. 4B). Moreover, after actinomycin D treatment, the RNA stability of SNHG17 in PC9/GR and A549/GR cells was higher than that in PC9 and A549 cells (Fig. 4C). Then, the expression of METTL3 in PC9, A549, PC9/GR and A549/GR cells were examined by qRT-PCR. Compared with that in PC9 and A549 cells, METTL3 expression was dramatically elevated in PC9/GR and A549/GR cells (Fig. 4D). To investigate whether METTL3 plays a role in m6A modification in PC9/GR and A549/GR cells, we detected the changes of m6A modification in METTL3-overexpressing PC9/GR and A549/GR cells. As shown in Fig. 4E, transfection of METTL3-overexpressing plasmids caused a significant upregulation of METTL3 in PC9/GR and A549/GR cells. Moreover, METTL3 overexpression strikingly promoted m6A modification in PC9/GR and A549/GR cells (Fig. 4F). MeRIP-qPCR also showed that METTL3 overexpression greatly elevated the m6A modification of lncRNA SNHG17 (Fig. 4G). Furthermore, RNA stability analysis demonstrated that METTL3 overexpression enhanced the stability of lncRNA SNHG17 (Fig. 4H). These results indicated that METTL3 increased the abundance of m6A modification and enhanced the stability of lncRNA SNHG17.

### LncRNA SNHG17 epigenetically inhibited LATS2 expression in PC9/GR and A549/GR cells

**Fig. 5:**
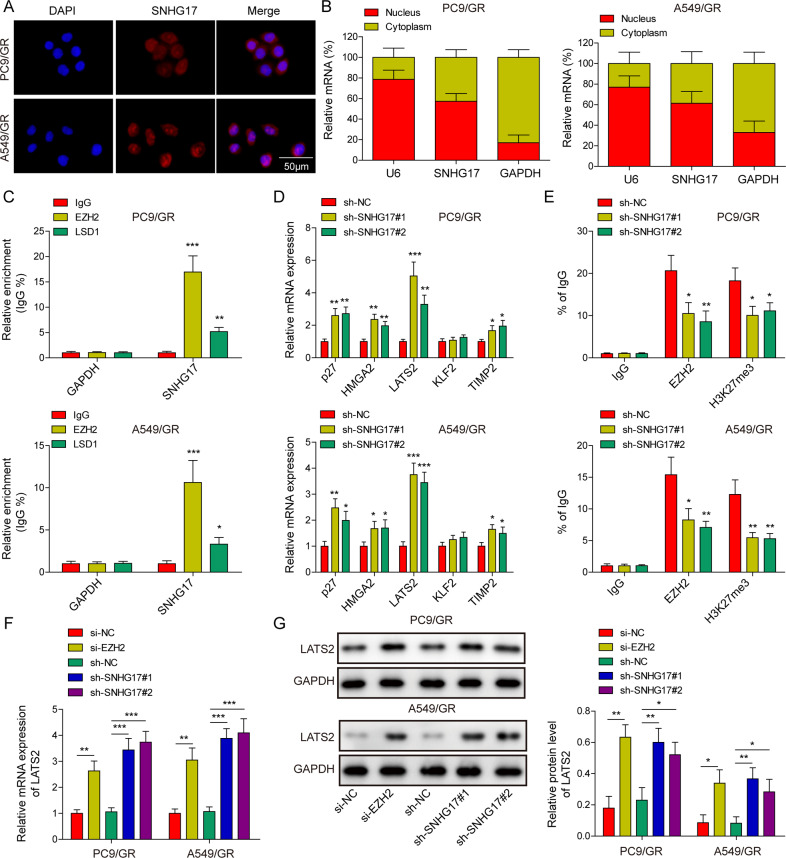
LncRNA SNHG17 epigenetically inhibited LATS2 expression in PC9/GR and A549/GR cells. **A**, **B** Distribution of lncRNA SNHG17 in the nucleus and cytoplasm in PC9/GR cells. **C** RIP-qPCR analysis was adopted to verify the interaction between lncRNA SNHG17 and EZH2 or LSD1. **D** Relative mRNA levels of p27, HMGA2, LATS2, KLF2 and TIMP2 were assessed by qRT-PCR in sh-NC, sh-SNHG17#1 and sh-SNHG17#2 transfected PC9/GR and A549/GR cells. **E** ChIP-qPCR was used to estimate the influences of sh-NC, sh-SNHG17#1 and sh-SNHG17#2 transfections on the binding levels of EZH2 and H3K27me3 on the LATS2 promoter. **F**, **G** Relative mRNA and protein expression of LATS2 was detected in EZH2- and lncRNA SNHG17-silenced PC9/GR and A549/GR cells. Data were representative images or were expressed as the mean ± SD of *n* = 3 experiments. *P < 0.05, **P < 0.01, ***P < 0.001.

To investigate the mechanism by which lncRNA SNHG17 regulates LUAD gefitinib resistance, FISH and subcellular fraction analysis were performed to detect its distribution in PC9/GR and A549/GR cells using a specific cy3-labelled lncRNA SNHG17 probe. The results showed that lncRNA SNHG17 was mainly located in the nucleus in PC9/GR cells (Fig. 5A). qRT-PCR also highlighted that lncRNA SNHG17 was mainly distributed in the nucleus in PC9/GR and A549/GR cells (Fig. 5B). These findings implied that lncRNA SNHG17 might act as a transcriptional regulator in the nucleus. Using RNA–protein interaction prediction (http://pridb.gdcb.iastate.edu/RPISeq/), we found that lncRNA SNHG17 potentially binds EZH2 and LSD1. RIP assay was subsequently performed to verify the interaction of lncRNA SNHG17 with EZH2 and LSD1. The results showed a great lncRNA SNHG17 showed a great abundance in the RNA complex precipitated by specific EZH2 or LSD1 antibodies (Fig. 5C). Then, we screened the expressions of potential targets of the lncRNA SNHG17/EZH2 axis, including P27, HMGA2, LATS2, KLF2 and TIMP2, in lncRNA SNHG17-silenced PC9/GR and A549/GR cells using qRT-PCR. Among these target genes, knockdown SNHG17 promoted the expression of LATS2 most significantly in both PC9/GR and A549/GR cells (Fig. 5D). We next investigated whether lncRNA SNHG17 could mediate the binding of EZH2 to the promoter region of LATS2 using a ChIP assay. The result indicated that lncRNA SNHG17 knockdown could reduce EZH2 and H3K27me3 occupation of the LATS2 promoter area in PC9/GR and A549/GR cells (Fig. 5E). In addition, the knockdown efficiency of siEZH2 in PC9 and A549 cells was verified by qRT-PCR (Fig. S3A). Then, knockdown of lncRNA SNHG17 or EZH2 dramatically enhanced the mRNA and protein expression of LATS2PC9/GR and A549/GR cells (Fig. 5F, G). Thus, these results suggested that lncRNA SNHG17 epigenetically inhibited the transcription of LATS2 by binding to EZH2 in vitro.

### EZH2 or lncRNA SNHG17 overexpression reversed the influences of LATS2 on PC9/GR and A549/GR cell proliferation and apoptosis

**Fig. 6:**
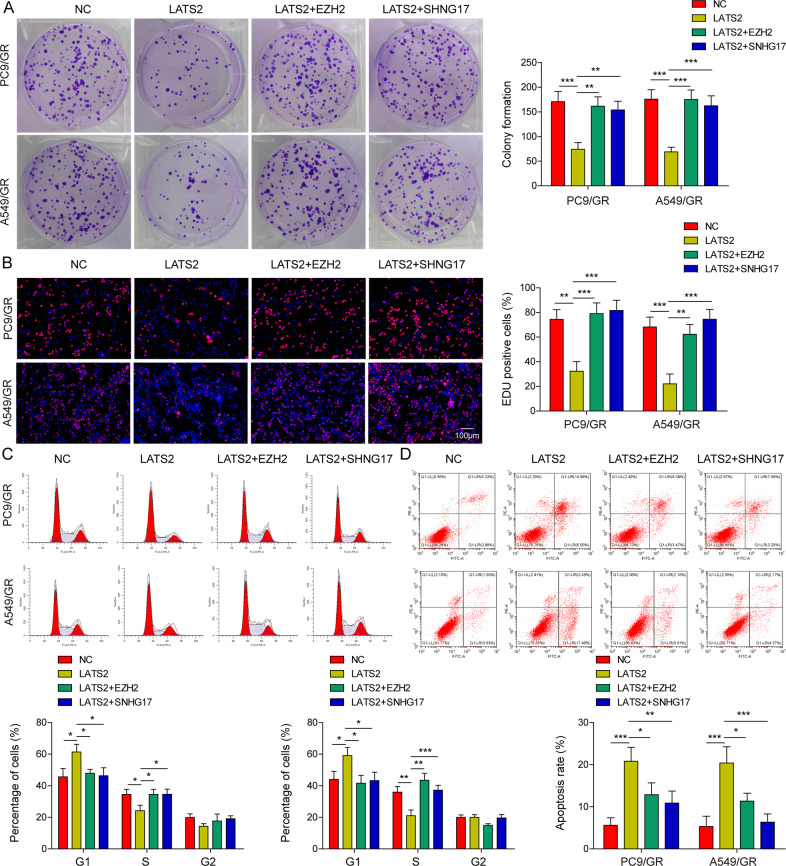
Overexpression of EZH2 or lncRNA SNHG17 reversed the influences of LATS2 on PC9/GR and A549/GR cell proliferation and apoptosis. PC9/GR and A549/GR cells were transfected with LATS2 alone or in combination with EZH2 or lncRNA SNHG17. **A** Colony formation and **B** EdU staining assays were employed to detect PC9/GR and A549/GR cell proliferation. Flow cytometry analysis was adopted to assess PC9/GR and A549/GR cell **C** cycle and **D** apoptosis. Data were representative images or were expressed as the mean ± SD of *n* = 3 experiments. *P < 0.05, **P < 0.01, ***P < 0.001.

To study the function of lncRNA SNHG17/EZH2/LATS2 in gefitinib resistance in LUAD, rescue assays were performed in PC9/GR and A549/GR cells. Then, LATS2-overexpressing vectors were transfected or cotransfected with an EZH2- or lncRNA SNHG17-overexpressing vector into PC9/GR and A549/GR cells. According to qRT-PCR analysis, the overexpression of LATS2 or/and EZH2 did not affect the expression of SNHG17, while SNHG17 transfection increased the expression of SNHG17 (Fig. S3B). The western blot analysis results indicated that the overexpression of LATS2 or/and SNHG17 did not affect the protein level of EZH2 (Fig. S3C). Moreover, LATS2 transfection elevated the protein level of LATS2, which could be reversed by the overexpression of EZH2 or SNHG17 (Fig. S3C). Colony formation and EdU staining assays demonstrated that LATS2 overexpression markedly repressed PC9/GR and A549/GR cell proliferation, while the inhibitory effects on cell proliferation could be reversed by the cotransfection of LATS2 and EZH2 or lncRNA SNHG17 (Fig. 6A, B). Flow cytometry analysis showed that LATS2 overexpression arrested the cell cycle and promoted cell apoptosis in PC9/GR and A549/GR cells, while cotransfection of LATS2 and EZH2 or lncRNA SNHG17 rescued these phenotypes (Fig. 6C, D). These findings indicated that overexpression of EZH2 or lncRNA SNHG17 reversed the regulatory influences of LATS2 on PC9/GR and A549/GR cell proliferation and apoptosis.

### Overexpression of EZH2 or lncRNA SNHG17 reversed the influences of LATS2 on PC9/GR and A549/GR cell migration, invasion and EMT

**Fig. 7:**
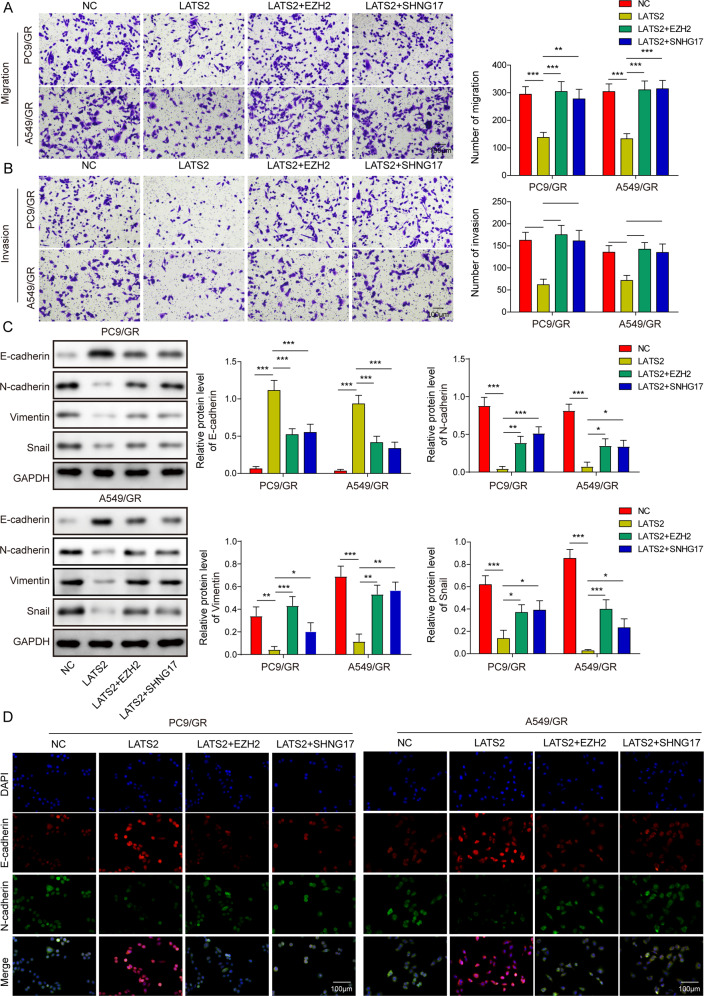
Overexpression of EZH2 or lncRNA SNHG17 reversed the influences of LATS2 on PC9/GR and A549/GR cell migration, invasion and EMT. PC9/GR and A549/GR cells were transfected with LATS2 alone or in combination with EZH2 or lncRNA SNHG17. **A**, **B** Transwell chambers with/without Matrigel were used to estimate PC9/GR and A549/GR cell migration and invasion, respectively. **C** Western blot analysis of E-cadherin, N-cadherin, Vimentin and Snail in treated PC9/GR and A549/GR cells. **D** Immunofluorescence staining analysis of E-cadherin and N-cadherin in treated PC9/GR and A549/GR cells. Data were representative images or were expressed as the mean ± SD of *n* = 3 experiments. *P < 0.05, **P < 0.01, ***P < 0.001.

Additionally, transwell assay indicated that LATS2 overexpression repressed the migratory and invasive abilities of PC9/GR and A549/GR cells, while cotransfection of LATS2 and EZH2 or lncRNA SNHG17 rescued these phenotypes (Fig. 7A, B). Additionally, western blot and immunofluorescence staining assays suggested that LATS2 overexpression increased E-cadherin levels and decreased N-cadherin, Vimentin and Snail levels, while these protein alterations caused by LATS2 overexpression could be reversed by the cotransfection of EZH2 or lncRNA SNHG17 (Fig. 7C, D). These findings indicated that overexpression of EZH2 or lncRNA SNHG17 reversed the influences of LATS2 on PC9/GR and A549/GR cell migration, invasion and EMT.

### LncRNA SNHG17 knockdown overcame gefitinib resistance in vivo

**Fig. 8:**
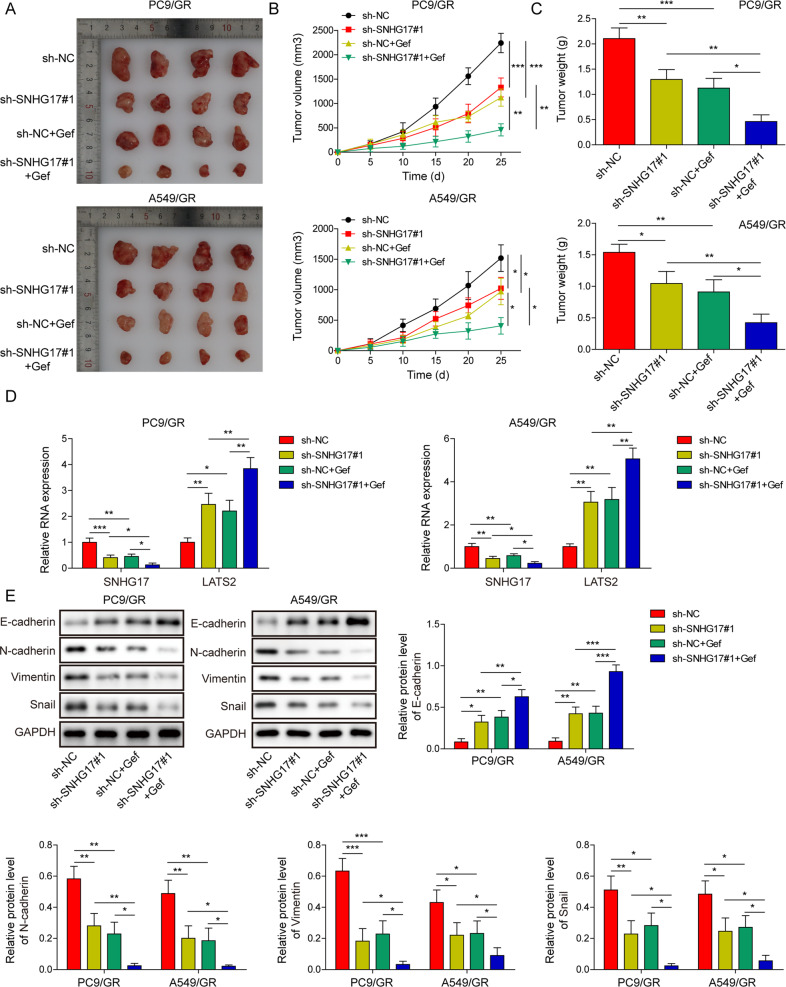
LncRNA SNHG17 knockdown overcame gefitinib resistance in vivo. **A** sh-NC- or sh-SNHG17-transfected PC9/GR and A549/GR cells were inoculated into nude mice. After 4 days of inoculation, the mice were treated with gefitinib (oral gavage, 5 days per week at 25 mg/kg). **B** Tumour volumes were measured every 4 days, and **C** tumour weights were measured after removal. **D** Relative expression of lncRNA SNHG17 and LATS2 mRNA in xenograft tumours was examined by qRT-PCR. **E** Protein expression of E-cadherin, N-cadherin, Vimentin and Snail in xenograft tumours was examined by western blot. The data were shown as representative images or were expressed as the mean ± SD of *n* = 8 experiments. *P < 0.05, **P < 0.01, ***P < 0.001.

PC9/GR and A549/GR cells stably transfected with sh-NC or sh-SNHG17#1 were inoculated into nude mice (male, 6 weeks old). After 4 days of inoculation, the mice were treated with gefitinib (oral gavage, 5 days per week at 25 mg/kg). After 28 days of inoculation, all mice developed tumours. The results indicated that lncRNA SNHG17 knockdown or treatment with gefitinib could decrease the tumour volume and weight. More importantly, with gefitinib treatment, tumours formed by cells infected with sh-SNHG17#1 were smaller than those formed by cells infected with sh-NC (Fig. 8A–C), suggesting that lncRNA SNHG17 knockdown enhanced the sensitivity of LUAD cells to gefitinib in vivo. The expression of LATS2 in tumours from each group was examined by qRT-PCR. LncRNA sSNHG17 knockdown or gefitinib treatment caused a remarkable upregulation of LATS2 in xenograft tumours tissues, while this effect was further strengthened by SNHG17 silencing and gefitinib co-treatment (Fig. 8D). Moreover, we found that lncRNA SNHG17 knockdown or treatment with gefitinib impaired the EMT process, as shown by the upregulation of E-cadherin and the downregulation of N-cadherin, Vimentin and Snail. More importantly, this phenotype was further enhanced in sh-SNHG17 plus the gefitinib group (Fig. 8E). These findings indicated that lncRNA SNHG17/EZH2/LATS2 promoted gefitinib-resistant LUAD tumorigenesis in vivo.

## Discussion

Gefitinib is currently used as a first-line treatment for lung cancer patients harbouring EGFR mutations [29, 30]. Unfortunately, the efficacy of gefitinib in patients with lung cancer may decline over the course of treatment due to the acquired gefitinib resistance in lung cancer tumours [31]. However, the mechanism of gefitinib resistance in lung cancer patients remains unclear. Herein, we reported an upregulation of lncRNA SNHG17 in gefitinib-resistant LUAD tissues and cells, implying a regulatory role of lncRNA SNHG17 in acquired gefitinib resistance in LUAD. Functional experiments showed that lncRNA SNHG17 silencing caused significant repression of LUAD cell growth and enhanced the gefitinib sensitivity of LUAD cells. Mechanistically, lncRNA SNHG17 could epigenetically repress LATS2 by recruiting EZH2 to the LATS2 promoter. Moreover, METTL3 was found to increase the abundance of m6A modification and enhance the stability of lncRNA SNHG17.

Abundant evidence suggests that lncRNAs can regulate drug resistance in human lung cancer cells through multiple mechanisms [32, 33]. The role of lncRNA SNHG17 in lung cancer was first reported by Xu Tianwei et al. in 2019. By analysing data from The Cancer Genome Atlas (TCGA), the authors found that SNHG17 was upregulated in NSCLC and acted as an oncogene in NSCLC [34]. Recently, lncRNA SNHG17 expression was also found to be elevated in LUAD cell lines, and evidence indicated that lncRNA SNHG17 can trigger the progression of LUAD by targeting miR-485-5p and increasing the levels of Wnt ligand secretion mediators [15]. Consistently, we confirmed the upregulation of lncRNA SNHG17 in LUAD; moreover, SNHG17 expression was higher in gefitinib-resistant LUAD than in gefitinib-sensitive LUAD. In subsequent functional assays, we demonstrated that lncRNA SNHG17 knockdown could suppress LUAD cell proliferation, invasion, migration and EMT while promoting LUAD cell apoptosis.

Studies have shown that m6A modification plays an important role in regulating biological activities and inactivity in lung cancer cells [35]. For example, ALKBH5 (an m6A demethylase) has been shown to inhibit the proliferation and migration of NSCLC cells by inhibiting YAP through the miR-107/LATS2 pathway [36]. METTL3 was reported to enhance cisplatin resistance in NSCLC by inducing YAP methylation to increase mRNA stability [37]. In this study, we found that METTL3 increased the abundance of m6A modification in lncRNA SNHG17, thus increasing the stability of lncRNA SNHG17. Thus, we inferred that the dysregulation of SNHG17 in LUAD might be correlated to METTL3.

In general, lncRNAs can regulate gene expression at various levels including chromatin remodelling, transcriptional regulation and post-transcriptional processing. According to existing evidence, polycomb repressive complex 2 (PRC2) is the most studied chromatin modifier recruited and regulated by lncRNAs [38]. PRC2, mainly composed of EZH2, SUZ12 and EED, is one of the important complexes of the PcG complex and mainly catalyzes H3K27me3 to induce gene silencing [39]. Accumulating studies have shown that abnormal expression, deletion or mutation of lncRNA and PRC2 are closely related to the occurrence and development of cancer [40]. Notably, EZH2 is the core subunit of the PRC2 complex, which can bind to lncRNA in lung cancer cells to regulate the expression of targeted genes [41, 42]. As previous reported, lncRNA SNHG17 was also demonstrated to facilitate gastric cancer progression through the inhibition of p15 and p57 by directly binding to EZH2 [14]. Moreover, the lncRNA SNHG17/EZH2/p57 axis was reported to exhibit promoting effects on colorectal cancer progression [11]. Additionally, EZH2 inhibition could reverse gefitinib resistance in lung cancer cells [43]. Thus, we speculated whether a similar mechanism existed in LUAD. Through a series of molecular validation, we confirmed the interaction relationship between SNHG17 and EZH2, which is similar to previous work [14]. Moreover, several targets regulated by SNHG17/EZH2 axis were found, such as LATS2, p27, HMGA2 and TIMP2. Whereas, the effect of these target genes on the biological function mediated by the SNHG17/EZH2 signal axis remains unclear.

As an important member of the LATS tumour inhibitor family, LATS2 has been reported to affect cellular homoeostasis, and low LATS2 expression has been found in various human cancers [44, 45]. LATS2 overexpression was demonstrated to suppress lung cancer cell proliferation and induce NSCLC cell cycle arrest and apoptosis [46, 47]. In this study, LATS2 overexpression was revealed to induce LUAD cell cycle arrest and apoptosis and repress gefitinib-resistant LUAD cell migration, invasion, and EMT. Rescue assays showed that the overexpression of EZH2 or lncRNA SNHG17 could block the impacts of LATS2 on gefitinib-resistant LUAD cells. These findings suggested that the lncRNA SNHG17/EZH2/LATS2 axis plays a key role in gefitinib-resistant LUAD progression. Furthermore, lncRNA SNHG17 knockdown was found to enhance gefitinib sensitivity in LUAD cells in vivo.

**Fig. 9:**
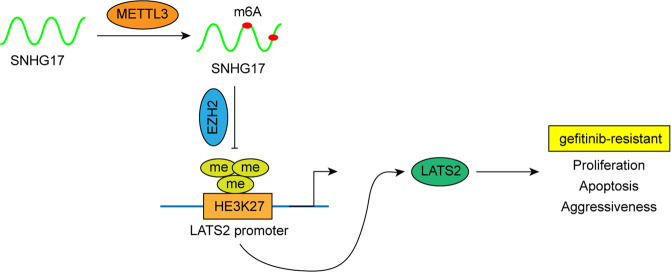
Mechanisms by which lncRNA SNHG17 regulates gefitinib resistance in LUAD. LncRNA SNHG17 facilitated gefitinib resistance by epigenetically inhibiting LATS2 transcription through the recruitment of EZH2.

In summary, our findings suggested that the m6A methyltransferase METTL3-induced lncRNA SNHG17 promotes gefitinib resistance in LUAD by epigenetically repressing LATS2 by recruiting EZH2 to the LATS2 promoter (Fig. 9), suggesting that the lncRNA SNHG17/EZH2/LATS2 axis might be a potential therapeutic target for gefitinib-resistant LUAD patients.

## Supplementary information

aj-checklist

Figure. S1

Figure. S2

Figure. S3

Figure.S1-3 legend

Language Editing Certificate

## Data Availability

The datasets used or analyzed during the current study are available from the corresponding author on reasonable request.
