# Supplementary figures and images for: m6A methyltransferase METTL3-induced lncRNA SNHG17 promotes lung adenocarcinoma gefitinib resistance by epigenetically repressing LATS2 expression
Source: Cell Death Dis. 2022 Jul 28;13(7):657. doi: 10.1038/s41419-022-05050-x (PMC9334586; doi:10.1038/s41419-022-05050-x)

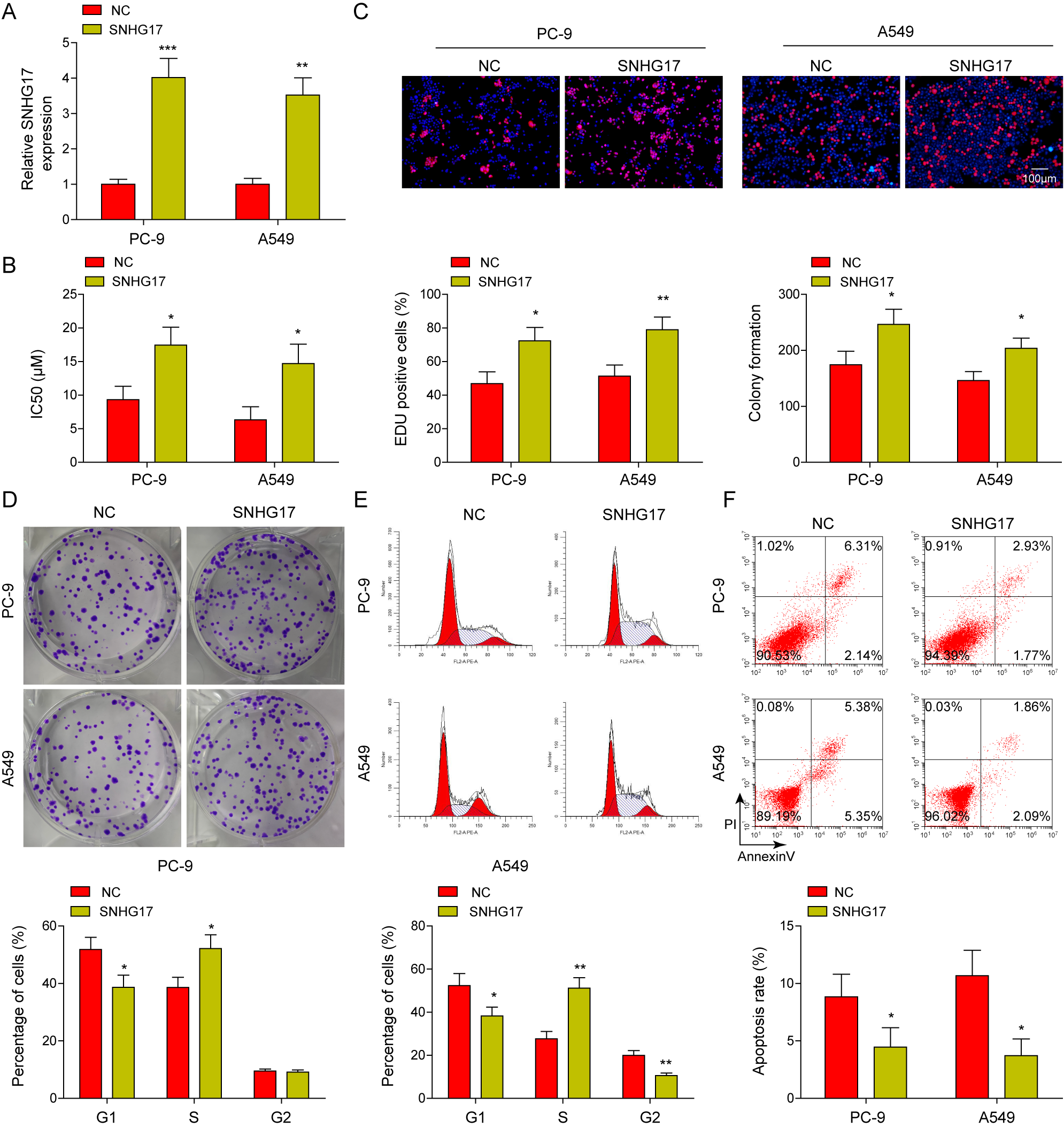

Supplement: Supplementary file 2 — Figure. S1 [file 41419_2022_5050_MOESM2_ESM.tif]

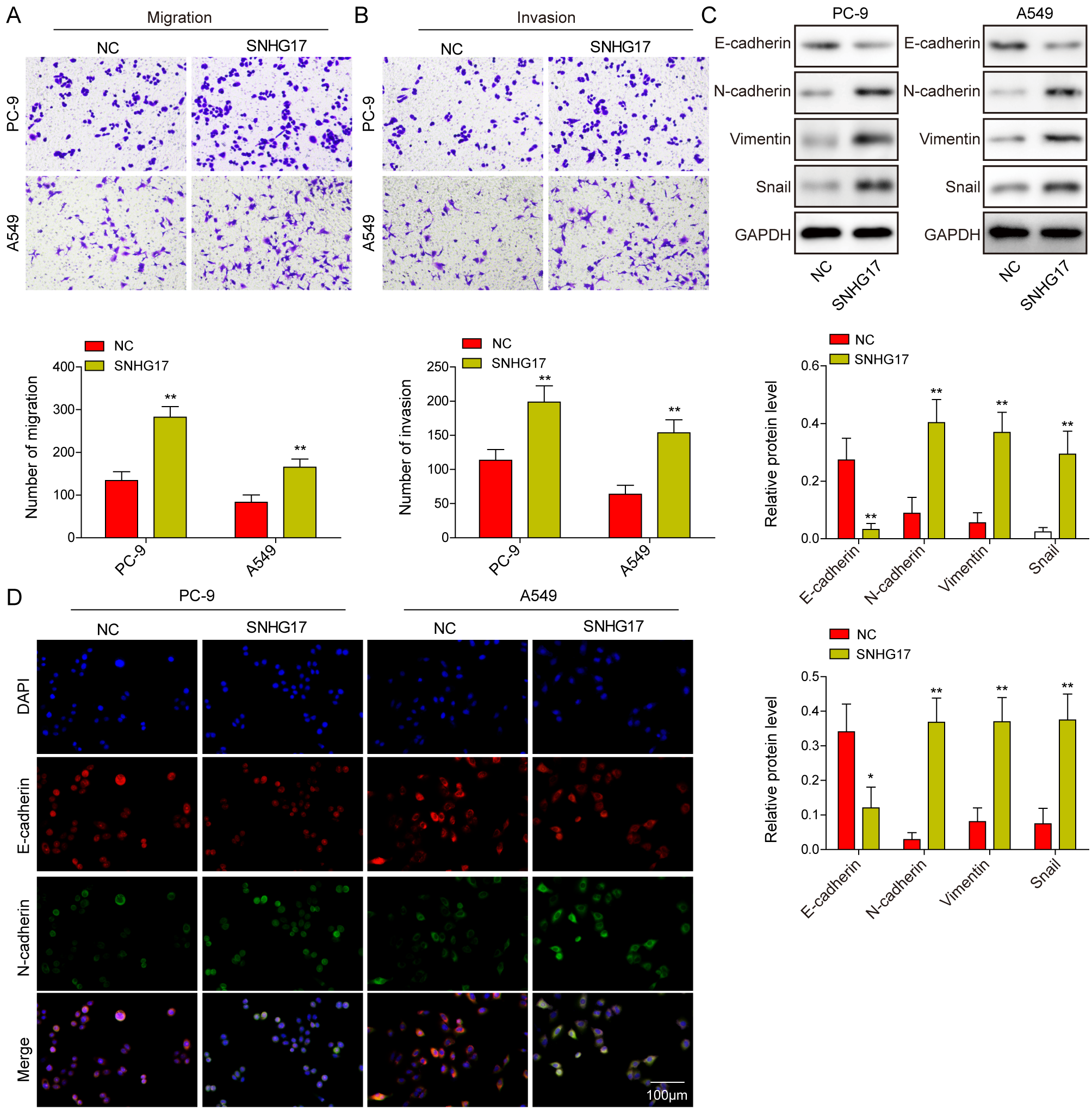

Supplement: Supplementary file 3 — Figure. S2 [file 41419_2022_5050_MOESM3_ESM.tif]

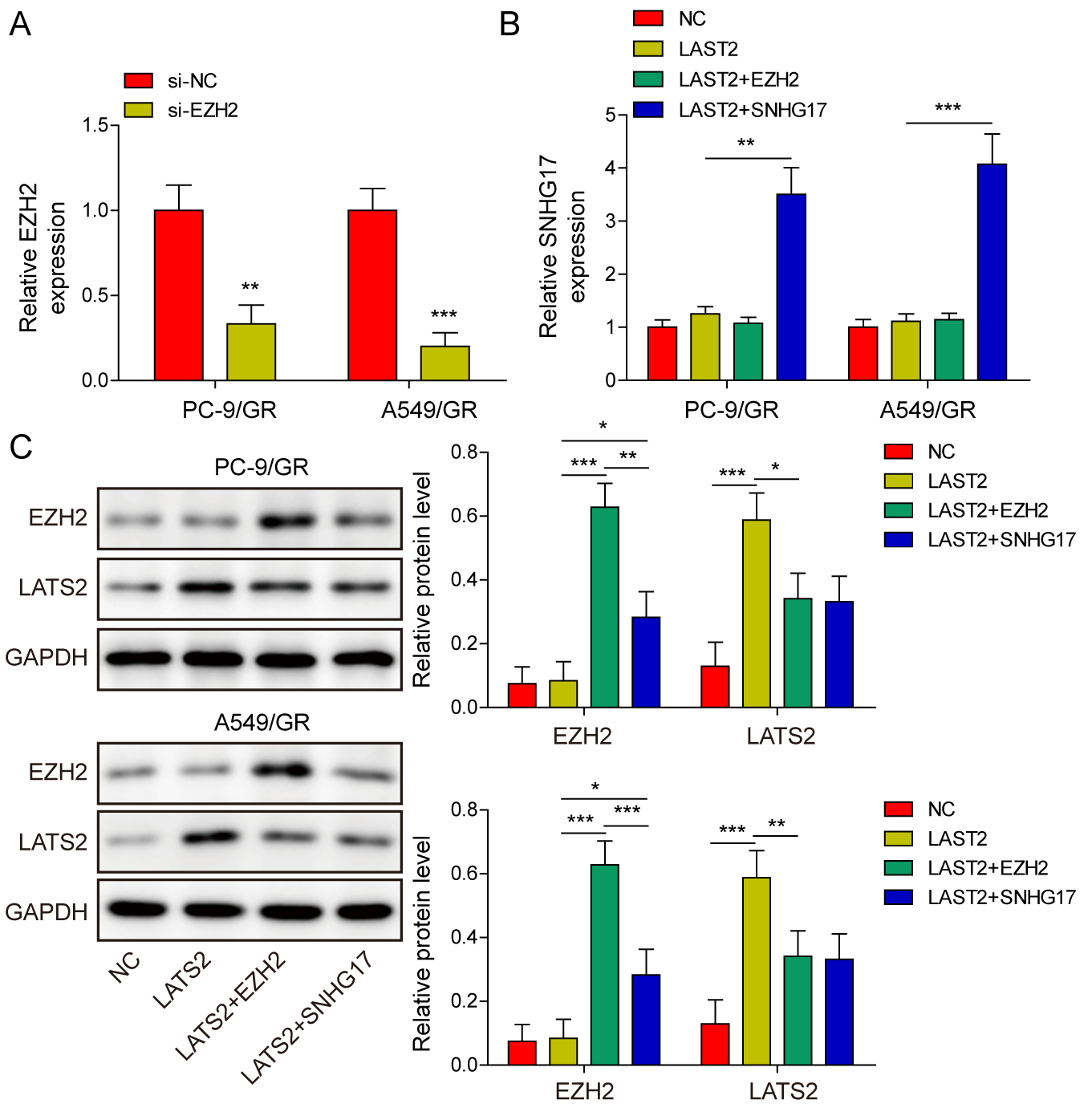

Supplement: Supplementary file 4 — Figure. S3 [file 41419_2022_5050_MOESM4_ESM.tif]
