# Supplementary material for: m6A methyltransferase METTL3-induced lncRNA SNHG17 promotes lung adenocarcinoma gefitinib resistance by epigenetically repressing LATS2 expression
Source: Cell Death Dis. 2022 Jul 28;13(7):657. doi: 10.1038/s41419-022-05050-x (PMC9334586; doi:10.1038/s41419-022-05050-x)
Supplement: Supplementary file 5 — Figure.S1-3 legend [file 41419_2022_5050_MOESM5_ESM.docx]

**Figure. S1.** LncRNA SNHG17 overexpression promoted LUAD cell proliferation while inhibited LUAD cell apoptosis. **(A)** Verification of the overexpression efficiency of pcDNA3.1-SNHG17 in PC9 and A549 cells using qRT-PCR. **(B)** CCK-8 assay was performed to determine the IC_50_ value of PC9 and A549 cells to gefitinib after transfection with pcDNA3.1-SNHG17 and empty vector. **(C and D)** Effects of lncRNA SNGH17 overexpression on cell proliferation were assessed by EdU staining and colony formation assay. **(E and F)** Flow cytometry analysis of cell cycle and apoptosis in pcDNA3.1-SNHG17 and empty vector transfected PC9 and A549 cells.

**Figure. S2.** LncRNA SNHG17 overexpression promoted LUAD cell migration, invasion and EMT. **(A and B)** Effects of lncRNA SNHG17 overexpression on LUAD cell migration and invasion were assessed by transwell assay. **(C)** Western blot analysis of E-cadherin, N-cadherin, Vimentin, and Snail in lncRNA SNHG17 overexpressed PC9 and A549 cells. **(D)** Immunofluorescence analysis of E-cadherin and N-cadherin in lncRNA SNHG17 overexpressed LUAD cells.

**Figure. S3. Relative EZH2 and SNGH17 expression in treated LUAD cells. (A)** Knockdown efficiency verification of siEZH2 in PC9 and A549 cells using qRT-PCR. **(B)** Relative expression analysis of SNG17 in LATS2, EZH2 or/and SNHG17 overexpressed LUAD cells using qRT-PCR. **(C**) Western blot analysis of EZH2, LATS2 in LATS2, EZH2 or/and SNHG17 overexpressed LUAD cells.
