# Supplementary material for: m6A methyltransferase METTL3-induced lncRNA SNHG17 promotes lung adenocarcinoma gefitinib resistance by epigenetically repressing LATS2 expression
Source: Cell Death Dis. 2022 Jul 28;13(7):657. doi: 10.1038/s41419-022-05050-x (PMC9334586; doi:10.1038/s41419-022-05050-x)
Supplement: Supplementary file 6 — Language Editing Certificate [file 41419_2022_5050_MOESM6_ESM.pdf]

Editing Certificate

This document certifies that the manuscript

m6A methyltransferase METTL3-induced lncRNA SNHG17 promotes lung adenocarcinoma gefitinib resistance by epigenetically repressing LATS2 expression

prepared by the authors

Heng Zhang

was edited for proper English language, grammar, punctuation, spelling, and overall style by one or more of the highly qualified native English speaking editors at AJE.

This certificate was issued on **April 27, 2022** and may be verified on the AJE website using the verification code **5D2D-44FE-4921-70F9-9373**.

Neither the research content nor the authors' intentions were altered in any way during the editing process. Documents receiving this certification should be English-ready for publication; however, the author has the ability to accept or reject our suggestions and changes. To verify the final AJE edited version, please visit our verification page at aje.com/certificate. If you have any questions or concerns about this edited document, please contact AJE at support@aje.com.

AJE provides a range of editing, translation, and manuscript services for researchers and publishers around the world.
For more information about our company, services, and partner discounts, please visit aje.com.
